# Formulation and *In-vitro *Evaluation of Tretinoin Microemulsion as a Potential Carrier for Dermal Drug Delivery 

**Published:** 2013

**Authors:** Seyed Alireza Mortazavi, Sanaz Pishrochi, Zahra Jafari azar

**Affiliations:** a *Department of Pharmaceutics, School of Pharmacy, Shahid Beheshti University of Medical Sciences, Tehran, Iran.*; b *Department of Pharmaceutics, Pharmaceutical Sciences Branch, Islamic Azad University, Tehran, Iran. *

**Keywords:** Tretinoin, Dermal absorption, Microemulsion, Drug release, Formulation

## Abstract

In this study, tretinoin microemulsion has been formulated based on phase diagram studies by changing the amounts and proportions of inactive ingredients, such as surfactants, co-surfactants and oils. The effects of these variables have been determined on microemulsion formation, particle size of the dispersed phase and release profile of tretinoin from microemulsion through dialysis membrane. In released studies, static Franz diffusion cells mounted with dialysis membrane were used. Sampling was conducted every 3 h at room temperature over a period of 24 h. The amount of released drug was measured with UV-spectrophotometer and the percentage of drug released was calculated. Based on the results obtained, the oil phase concentration had a proportional effect on particle size which can consequently influence on drug release. The particle size and the amount of released drug were affected by the applied surfactants. The components of the optimized microemulsion formulation were 15% olive oil, 12% propylene glycol (as co-surfactant), 33% Tween^®^80 (as surfactant) and 40% distilled water, which was tested for viscosity and rheological behavior. The prepared tretinoin microemulsion showed pseudoplastic-thixotropic behavior. The profile of drug release follows zero order kinetics. The optimized tretinoin microemulsion showed enhanced *in-vitro *release profile compared to the commercial gels and creams.

## Introduction

Drug delivery to or via skin has provided an effective route for local or systemic administration of therapeutically active agents. However, skin and in particular, stratum corneum provides an efficient protective barrier for drug absorption. Stratum corneum (horny layer) has a thickness of about 15-20 μm, and is a layer of compressed, overlapping keratinized cells that form a flexible, tough and coherent membrane. This layer contains dead cells with keratin filaments in a matrix of proteins with lipids and water-soluble substances. The thickness and penetration properties of stratum corneum depend upon its hydration, which normally contains around 20% water (1, 2). 

Acne vulgaris is the most common disorder treated by dermatologists. It is a self-limited chronic inflammatory disease of pilosebaceous units characterized by the formation of open and closed comedones, pustules, papules and cysts. Acne vulgaris commonly occurs on the face and to a lesser extent on the back, chest and shoulders. Although acne vulgaris is primarily a disease of adolescence, it may persist or even become chronic during adulthood. Near 80% of these cases are within the age of 11-30 years. Acne can be psychosocially malignant (3).

Acne has a very complex pathophysiology. Most common abnormal findings in acne pathology includes: increased secretion of sebum by sebaceous glands, epithelial hyper keratinization of sebaceous follicules, skin colonization by propionibacterium acne (*P. acne*) and inflammation. *P. acne *has a crucial role in acne, so antibiotics active against this organism, topical or systemic are the most commonly used drugs in acne treatment. Isolates of *P. acne *resistant to one or more anti-acne antibiotics are being frequently reported and emergence of such strains can be associated with therapeutic failure. One of the best treatments for antibiotic resistant *P. acne *strains is the use of non-antibiotic active ingredients. For this purpose, tretinoin can be one of the best choices. Tretinoin is one of the most effective medications for acne and is a derivative of retinol. It can be used in the treatment of various skin disorders such as acne, photo-aging and severe conditions like psoriasis and squamous cell carcinoma (4-6).

Usually, oral administration of isotretinoin, an anti-acne agent, may cause side effects such as cheilitis, conjunctivitis, irritation, and increased erythrocyte sedimentation rates. To avoid these side effects, blood lipid level tests, liver function tests and hematological tests may be advised, before or during the consumption.

Transdermal drug delivery systems of tretinoin can avoid the systemic side effects of its oral dosage forms and help to achieve a better therapeutic effect. Transdermal drug delivery uses the skin as an alternative route for the delivery of systemically active drugs and has several advantages, over oral drug administration (7). Transdermal drug delivery system (TDDS) offer a number of advantages like bypassing the first pass effect, reducing frequency of administration, potentially decreasing side effects, improved patient compliance sustaining drug delivery and interruption or termination of treatment when necessary (8, 9).

In recent decades, for skin care and the topical treatment of dermatological disease, a wide choice of vehicles ranging from solids to semisolids and liquid preparations is available to clinicians and patients (10-13). Low percutaneous absorption of active ingredients from these bases, can lead to low drug concentrations at the absorption site, which can result in a lower bioavailability (11). Attempts to increase the percutaneous absorption of these compounds, has been the main problem of these dosage forms. One way to solve this problem is to use microemulsion formulations, composed of the active drug, aqueous phase, oil phase, surfactant and co-surfactant. These drug delivery systems are thermodynamically stable, transparent, single phase and also optically isotropic solution with a droplet diameter usually within the range of 10-100 nm (14-20). Although different reports have been published on microemulsions, but so far only a few commercial microemulsion-based products have reached the market which could be due to the physical instability of the active ingredient present within the formulation.

In a study by Zabka and co-workers the invitro release efficacy of diclofenac sodium and indomethacin across human skin was ranked higher than the other topical dosage forms tested (15). In another study several co-surfactants (isopropyl alcohol and propylene glycol) were found to increase the penetration of diclofenac sodium across skin membrane (21).

El laithy and El-Shaboury found that when the particle size decreases, the number of vesicles that can interact on a fixed area of stratum corneum will increase so it seems the particle size of the microemulsion may also affect on its efficiency (11).

Moghimipour and colleagues demonstrated that physicochemical properties and *in-vitro *release of tretinoin microemulsion were dependent upon the contents of surfactant/co-surfactant, water and oil percentage which were used in formulations (18). Finally, in a study by Suthar *et al. *it was found that the use of octanol as a co-surfactant could be useful in the preparation of tretinoin microemulsion based gel, in order to improve the drug release profile of the active ingredient in comparison with tretinoin commercial cream (6).

The present study was designed to investigate the influence of various surfactants, co-surfactants and oils, when used in different proportions, on the formation of topical tretinoin microemulsion, particle size and tretinoin release profile from microemulsion through dialysis membrane. Furthermore, the release profile of these formulations has been compared with commercial gels and creams.

## Experimental

Tretinoin was supplied by Roche Co., Switzerland. Olive oil and castor oil were purchased from Sigma-Aldrich Chemical Co,. Germany. glyceryl Stearate was purchased from Serva Co., USA. Tween^®^20, Tween^®^40, Tween^®^80, stearyl alcohol, Span^®^20, Span^®^80, PG (propylene glycol), isopropanol, PEG 4000 (polyethylene glycol 4000), PEG 6000 (polyethylene glycol 6000), Isopropyl myristate was purchased from Merck Chemical Co., Germany. Ethanol (96%) was supplied by Arak teb company., Iran. All materials were used as received.

*Methods*

In this study, tretinoin microemulsions have been formulated by changing the amounts and proportions of inactive ingredients, including surfactants, co-surfactants and oils. Tretinoin microemulsions were prepared by mixing appropriate amounts of surfactant, co-surfactant, and aqueous phase with varying ratio as described in Table 1. Then, 0.005 g of tretinoin was added. Finally, appropriate amounts of oil was added to the formulations dropwise and mixed at 2000 rpm for 4 h at room temperature. The end point of each preparation was the point before the formulation became turbid or cloudy. The surfactants used in this study included Tween^®^20, Tween^®^40, Tween^®^80, glyceryl stearate, stearyl alcohol, Span^®^20, Span^®^80 and the co-surfactant used included PG, ethanol, isopropanol, PEG 4000, PEG 6000. Formulations were made in three groups by the aid of different oils, such as olive oil, castor oil and isopropyl myristate.

**Table 1:** Composition of various microemulsion formulations prepared in this study

**Formulation code**	**Amount of oil phase (%)**	**Amount of surfactant and co-surfactant (%)**	**Amount of aqueous phase (%)**	**Surfactant : Co-surfactant ratio**	**Oil: Surfactant/Co-surfactant ratio**
**ME-1**	17	45	38	2.2:2.8	1.37:3.62
**ME-2**	15	45	40	2.2:2.8	1.25:3.75
**ME-3**	10	45	45	2.2:2.8	0.90:4.09
**ME-4**	17	45	38	3.1:1.9	1.37:3.62
**ME-5**	15	45	40	3.1:1.9	1.25:3.75
**ME-6**	10	45	45	3.1:1.9	0.90:4.09
**ME-7**	17	45	38	3.6:1.3	1.37:3.62
**ME-8**	15	45	40	3.6:1.3	1.25:3.75
**ME-9**	10	45	45	3.6:1.3	0.90:4.09
**ME-10**	17	45	38	4.4:0.6	1.37:3.62
**ME-11**	15	45	40	4.4:0.6	1.25:3.75
**ME-12**	10	45	45	4.4:0.6	0.90:4.09

After each preparation, the formulations were tested in terms of visual properties consist of appearance and texture. In each test a layer of microemulsion formulations prepared were placed between two glass slides. Formulations with transparent appearance and uniform texture were selected for the other tests.

Among all the products, microemulsions with the code of ME-7, ME-8, ME-9 were selected as the optimized formulations which were tested for pH, droplet size, tretinoin Assay, *in-vitro *drug release and rheological properties. Details of these tests have been described as follows.

*pH measurement*

The pH values of the prepared microemulsion formulations were measured by a pH meter (CH-8603 model, Mettler-Toledo AG, Switzerland), at 25 ± 1°C in triplets after the dilution of microemulsion at a ratio of 1:10 with distilled water.

*Particle size measurement*

The average droplet size of the prepared microemulsions was measured in triplicate at 25°C using Quidix Microparticle size analyzer (Scatterscope I model, Quidix Inc., South Korea).

*Viscosity measurement and evaluation of rheological properties*

The viscosities of the selected microemulsions were measured at room temperature in triplets, using a cone and plate Brookfield RVDV-Ш viscometer (Brookfield Inc., USA). 0.5 of the test sample was used in each test. Different shear rates and shear stresses were applied on the test samples, and the resulting rheogram was constructed to determine the rheological behavior and viscosities of the test samples.

*Drug release studies*

For the purpose of this study, a mixture of ethanol 96 and pH of 7.3 phosphate buffer solution (at a ratio of 1:2) was used as the receptor phase. Dialysis membrane was cut into small circular sections with a diameter of 6 cm and carefully placed between the receptor and donor compartments of the diffusion cell. To achieve a fully contact between dialysis membrane and receptor phase, air bubbles were left out. After placing the cell cap on the donor compartment, areas around the cell cap were tightly closed by parafilm. Then, in order to ensure that the dialysis membrane is fully hydrated, the receptor compartment was filled with 50 mL of the ethanol and pH of 7.3 phosphate buffer solution (receptor phase) and kept in contact for a period of 24 h at 4°C. Following this period, the receptor phase was completely removed and replaced with fresh receptor phase. Next, 1.0 g of the prepared tretinoin microemulsion was gently placed in contact with the dialysis membrane of the donor compartment, while the receptor compartment (50 mL) was filled with the mixture of ethanol 96 and pH of 7.3 phosphate buffer solution (at a ratio of 1:2). The temperature of cell assembly was maintained at 25 ± 2°C and contents of the receptor compartment were stirred using a magnetic stirrer at 100 rpm and sampling was taken over 24 h with 3 h intervals and the cell was refilled with equal volumes of fresh medium. The amount of tretinoin released into the receptor phase from the microemulsion formulation was then calculated by determining the UV absorbance of the samples removed at 335.5 nm, using Shimadzu 1650PC UV-Visible spectrophotometer. Next, the percentage of the released drug was calculated using a constructed calibration curve of tretinoin pure powder, which was found to be linear (r = 0.99998). The equation obtained was “absorbance = 0.156 x concentration -0.026”. All the studies were performed in triplicate.

In order to find out the mechanism of tretinoin release from the selected microemulsion formulations, the data obtained from *in-vitro *release studies were fitted to three kinetic equations:

a) Q_t_ = k_0_t (zero-order equation)

b) ln Q_t _= ln Q_0_ - k_1_. t (first-order equation)

c) Q_t_ = K. S. t^0.5^ = k_H_ . t^0.5^ (Higuchi equation based on Fickian diffusion)

Here, Q is the amount of drug release in time t, Q_0_ is the initial amount of drug in the nanoparticles, S is the surface area of the nanoparticle and k_0_, k_1_ and kH are rate constants of zero order, first order and Higuchi equation, respectively (22).

*Tretinoin assay*

Drug assay was performed using UV spectrophotometric method at λmax = 335.5 nm using Shimadzu 1650PC UV-Visible spectrophotometer. For this purpose, 1.0 g of tretinoin microemulsion was transferred to a 100 mL volumetric flask and diluted with ethanol and pH of 7.3 Phosphate buffer (at a ratio of 1:2) to volume. Next, the amount of tretinoin present within a microemulsion formulation was determined. The same calibration curve was used for drug release studies. These studies were also performed in triplicate.

*Statistical analysis*

Results were expressed as mean ± SD and the data were analyzed by SPSS 17.0 statistical software. A paired sample t-test was used to examine the statistical difference in the release profile between formulations. The value of p < 0.05 was considered as statistically significant.

## Results and Discussion

Tretinoin (All-trans-retinoinc acid) is one of the most effective medications for the treatment of moderate-to-severe photo-damaged facial skin such as acne, photo-aging and severe conditions like psoriasis and squamous cell carcinoma. The main effect of tretinoin in the treatment of acne is to reduce the size and the number of comedones. Tretinoin is commonly used in a concentration of 0.05% w/w, incorporated in a lotion or/and an o/w cream (23). Passage of drug molecules through the skin could be an important and rather troublesome stage in percutaneous drug delivery (24). In order to solve this problem and increasing the drug absorption through the skin, novel drug delivery systems were used.

Among the various transdermal drug delivery systems, microemulsions appear to be an appropriate dosage form used for increasing cutaneous delivery, improving thermodynamic stability, products appearance, physicochemical characteristics and enhancement of bioavailability. Topical tretinoin can cause major side effects often appearing in the form of scaling, erythema, burning and stinging (23, 25). The marketed products such as isotretinoin cream show significant skin irritation and systemic absorption, which is associated with side effects (26). To overcome this problems tretinoin is incorporated into microemulsion. Tretinoin-loaded microemulsion formulations can increase the drug release profile, hence minimize the irritation effects. This delivery system works by entrapping the drug in vesicles, which brings the medication more directly to the follicle for a better therapeutic response (27). Tretinoin is also very unstable to radiation. In this regard the use of microemulsion is considered very useful (28). Therefore, incorporation of tretinoin vesicles can protect drug against photo-degradation (29).

In this study, attempts were made to investigate the effects of various types and amounts of surfactants, co-surfactants and oils, on topical tretinoin microemulsion formation, particle size and tretinoin release profile from microemulsion through dialysis membrane. Previous tretinoin microemulsion phase diagram studies showed that formulations which are in o/w microemulsion regions could be identified as the optimum formulations for tretinoin microemulsions (6). The pseudoternary phase diagrams were constructed by the aid of previous studies to determine the microemulsion region and detect the possibility of microemulsion formation with different compositions of oil, surfactant, co-surfactant, and water (6, 18).

Table 1 shows the composition of various microemulsion formulations containing tretinoin and inactive ingredients, prepared in this study. Based on preliminary studies and construction of a ternary phase diagram, the formulations were made by changing the types and proportions of surfactants and co-surfactants. Microemulsions were prepared, using each surfactant alongside co-surfactants. Presence of the glycols family such as PEG 4000 and PEG 6000 resulted in a white appearance in the product and a solid texture in a range of 56-26% w/w. This could be explained because of their high molecular weight. In order to improve the visual properties of the formulations, this percentage was decreased to 12% w/w, which made no difference. Formulations containing ethanol and isopropanol (as co-surfactants) had low apparent viscosities, which showed phase separation after a week. The results showed that among all the co-surfactants, propylene glycol at a ratio of 3.6:1.3 can produce the most suitable viscosity and also the best texture.

The next excipient investigated was surfactants used to stabilize the emulsion. In the process of formulating the tretinoin microemulsion, it was observed that formulations containing different concentrations of Spans (Span^®^20 and Span^®^80) formed translucent microemulsions. This observation could be attributed to the fact that spans, based on their lypophilic nature, could not prepare the o/w microemulsion. It was found that, in case of formulations containing the same oil phase and co-surfactant at different concentrations, the concentration of the glyceryl stearate and stearyl alcohol (as a surfactant) increased the apparent viscosities escalated, accordingly. This phenomenon can be attributed to the fact that, at higher concentration of the glyceryl stearate and stearyl alcohol, due to the long chains length in their structures, three-dimensional structures could be formed which affect on the apparent viscosity.

The appearances and apparent viscosities of different tretinoin microemulsion formulations have been shown in Table 2.

**Table 2:** Physical appearances and apparent viscosities of tretinoin microemulsion formulations (ME-1 – ME-12).

**Formulation code**	**Physical appearance**	**Apparent viscosity**
**ME-1**	Cloudy, light yellow color	Low
**ME-2**	Translucent, light yellow color	Low
**ME-3**	Translucent, light yellow color	Low
**ME-4**	Translucent, light yellow color	Relatively low
**ME-5**	Translucent, light yellow color	Relatively low
**ME-6**	Translucent, light yellow color	Relatively low
**ME-7**	Slightly translucent,yellow color	Relatively high
**ME-8**	Transparent,yellow color	Relatively high
**ME-9**	Transparent,yellow color	Relatively high
**ME-10**	Transparent,yellow color	High
**ME-11**	Transparent,yellow color	High
**ME-12**	Transparent,yellow color	High

Formulations ME-1, ME-2, ME-3 could not prepare microemulsion system due to an unsuitable ratio of surfactant/co-surfactant and insufficient amounts of surfactant in the formulation. Formulations ME-10, ME-11, ME-12 showed high viscosities. This could be due to the fact that tweens - based on their hydrophilic nature and large number of polyoxyethylene groups available in their structure - tend to absorb the aqueous phase and increase the viscosity by reducing the free-water of the formulations.

From these studies, it was revealed that Tweens and propylene glycol with a ratio of 3.6:1.3 were the best surfactants and co-surfactant used, respectively, which resulted in the formation of transparent stable microemulsions.

Among the rest of the formulated microemulsions, formulations contained Tween^®^20, Tween^®^40 and Tween^®^80 showed suitable apparent viscosities as well as good physical appearances. Hence, they were used for further studies.

For this purpose first, the effect of oil phase in concentrations of 10, 15 and 17% w/w within the microemulsion, on tretinoin release through dialysis membrane and particle size were investigated (Table 3).

**Table 3:** A comparision between the mean particle size and drug release profile of formulations ME-7 – ME-9; (n = 3, mean ± standard deviation).

**Formulation code**	**Apparent viscosity**	**Visual appearance**	**Mean particle size (nm ± SD)**	**Amount of drug release (% ± SD)**		
				**After 8 (h)**	**After 12 (h)**	**After 24 (h)**
**ME-7**	Relatively low	Slightly translucent, yellow color	992 ± 39.27	17.44 ± 0.29	0.31 ± 26.81	0.26 ± 42.07
**ME-8**	Relatively low	Transparent, yellow color	319 ± 86.21	0.43 ± 34.76	0.37 ± 49.50	0.84 ± 82.07
**ME-9**	Relatively low	Transparent, yellow color	214 ± 52.34	0.36 ± 6.33	0.52 ± 9.76	0.19 ± 28.94

When using a concentration of 10% w/w oil phase in the microemulsion formulation, the apparent viscosity was found to be more than formulation containing 17% oil phase. The release profiles of tretinoin obtained from evaluating the prepared microemulsion formulations of ME-7, ME-8 and ME-9 have been presented in Table 3. It was observed that, generally increasing the surfactant to oil phase ratio from 3.62:1.37 to 4.09:0.90, could reduce the release of drug towards medium phase. As it can be seen in Table 3, the mean particle size of dispersed phase was found to be increased with the increase in the concentration of oil phase from ME-9 (10% w/w) to ME-7 (17% w/w). It has already been reported that particle size is directly proportional to the concentration of dispersed phase (30, 31-33). The results demonstrated that when the oil phase with a higher concentration was used, bigger droplets were formed with larger mean particle size and the formulation appeared slightly cloudy.

Next, in order to evaluate the influence of the oil types and different types of Tween on ME-8 properties for each type of oils, three kind of Tweens were tested (Table 4).

**Table 4:** The composition of selected tretinoin microemulsion formulations ME-8a, ME-8b and ME-8c based on various oils and surfactants (n = 3, mean ± standard deviation

**Formulation code**	**Type of Oil phase**	**Type of surfactant**	**Type of co-surfactant**	**pH**	**Particle size (nm ± SD)**
**ME-8a2**	Olive oil	Tween^®^20	PG	0.05 ± 6.23	31.25 ± 414
**ME-8a4**	Olive oil	Tween^®^40	PG	0.08 ± 6.54	117.09 ± 629
**ME-8a8**	Olive oil	Tween^®^80	PG	0.12 ± 6.22	86.21 ± 319
**ME-8b2**	Castor oil	Tween^®^20	PG	0.09 ± 6.48	6.18 ± 553
**ME-8b4**	Castor oil	Tween^®^40	PG	0.17 ± 6.62	41.67 ± 875
**ME-8b8**	Castor oil	Tween^®^80	PG	0.16 ± 6.41	29.71 ± 458
**ME-8c2**	Isopropyl myristate	Tween^®^20	PG	0.14 ± 6.33	93.46 ± 481
**ME-8c4**	Isopropyl myristate	Tween^®^40	PG	0.19 ± 6.27	71.32 ± 701
**ME-8c8**	Isopropyl myristate	Tween^®^80	PG	0.10 ± 6.46	84.39 ± 340

After preparing each formulation, the mean particle size of tretinoin microemulsion formulations in each group (a-c) was measured by the aid of microparticle size analyzer. Based on the results, among formulations in group (a) which contain olive oil as the oil phase, formulation ME-8a8 which contained Tween^®^80 (as a surfactant) showed a smaller particle size than formulations ME-8a2 and ME-8a4 which contained Tween^®^20 and Tween^®^40. In addition, formulations ME-8a2 and ME-8a4 did not release a sufficient amount of drug within 24 h.

Microemulsion formulations in group (b) were made of 15% w/w castor oil, 32.4% w/w Tween, 12.6% w/w PG and 40% distilled water. In this group only the particle size of formulation ME-8b8 was found to be within the acceptable limit. This is presumably due to the presence of castor oil in this formulation. On the other hand, in formulations ME-8b2 and ME-8b4 the presence of Tween^®^20 and Tween^®^40 could also result in large droplet size and hence a lower amount of drug release. It seems that the type of surfactant can influence the particle size and have a consequent impact on the extent of drug release from the prepared microemulsion base.

Microemulsion formulations in group (c) were made of isopropyl myristate as the oil phase. As can be clearly observed in the results obtained in Table 4, the mean particle size of ME-8c8 is in the range which is more acceptable for topical microemulsion drug delivery. When considering the drug release profile of group (c), formulation ME-8c8 was selected as the better formulation with a drug release of 74.15% . It seems that the type of surfactant is the reason of this difference.

Finally, in a comparison between three formulations ME-8a8, ME-8b8 and ME-8c8, the lowest mean particle size was observed in formulation ME-8a8, followed by formulations ME-8c8 and ME-8b8. Between these three formulations, formulation ME-8a8 which contained olive oil and Tween^®^80 showed a greater amount of drug release than formulation ME-8b8 and ME-8c8, which contained castor oil and isopropyl myristate. Formulation ME-8a8 was selected for complementary studies.

*Particle size analysis*

The mean droplet size was determined by the aid of Microparticle size analyzer. Microemulsion particle size was found to be increased with the increase in the concentration of the oil phase (Table 3). 

The mean particle size of selected microemulsion formulations ME-8a8, ME-8b8 and ME8c8 were 300-450 nm, which are suitable enough for topical microemulsion formulations (Table 4). As it can be seen, the particle size is increased by changing the types of the surfactants used. The mean particle size of the selected formulation ME-8a8 was 319 ± 86.21, which was selected as the best mean particle size.

*In-vitro release study*

**Figure 1:**
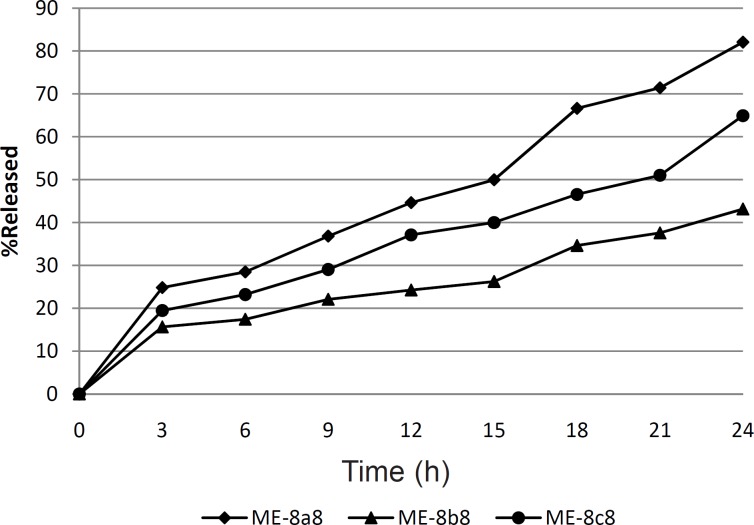
*In-vitro *drug release profile of ME-8a8, ME-8b8 and ME-8c8 through dialysis membrane (n = 3, SD < 5%).

Figure 1 shows the drug release profile from the three selected tretinoin microemulsion formulations. In this figure, tretinoin released from formulations ME-8a8, ME-8b8 and ME-8c8 were compared. It can be seen that drugs released from different microemulsion formulations are not the same. As can be seen in this figure, nearly 82.07% of the drug content of the ME-8a8 was released in 24 h. On the contrary, formulation ME-8b8 showed the lowest amount of drug released. This means that the addition of castor oil, alongside the Tween^®^80 cannot produce the same amount of drug release observed with formulation ME-8a8 which contained olive oil alongside Tween^®^80. Although the release profile of formulation ME-8c8 was not acceptable, it showed higher release than formulation ME-8b8.

Statistical analysis of the release profiles obtained among formulations ME-8a8, ME-8b8 and ME-8c8 showed a significant difference between the results obtained (p < 0.05). Therefore, among three selected formulations tested for determination of drug release profile, only the formulation ME-8a8 could release the most drug content throughout 24 h period, which was the most desirable as compared to other formulations.

**Figure 2:**
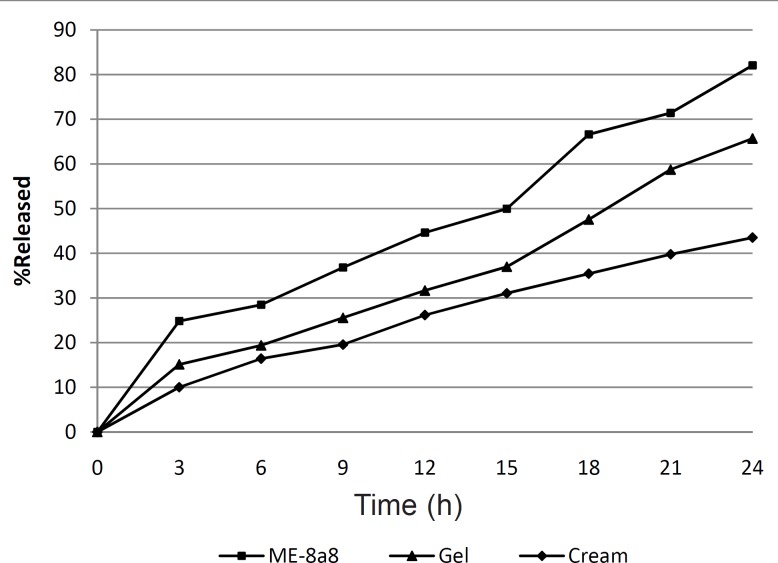
*In-vitro *drug release profile of TME4,Tretinoin gel and Tretinoin cream through dialysis membrane (n = 3, SD < 5%).

**Figure 3:**
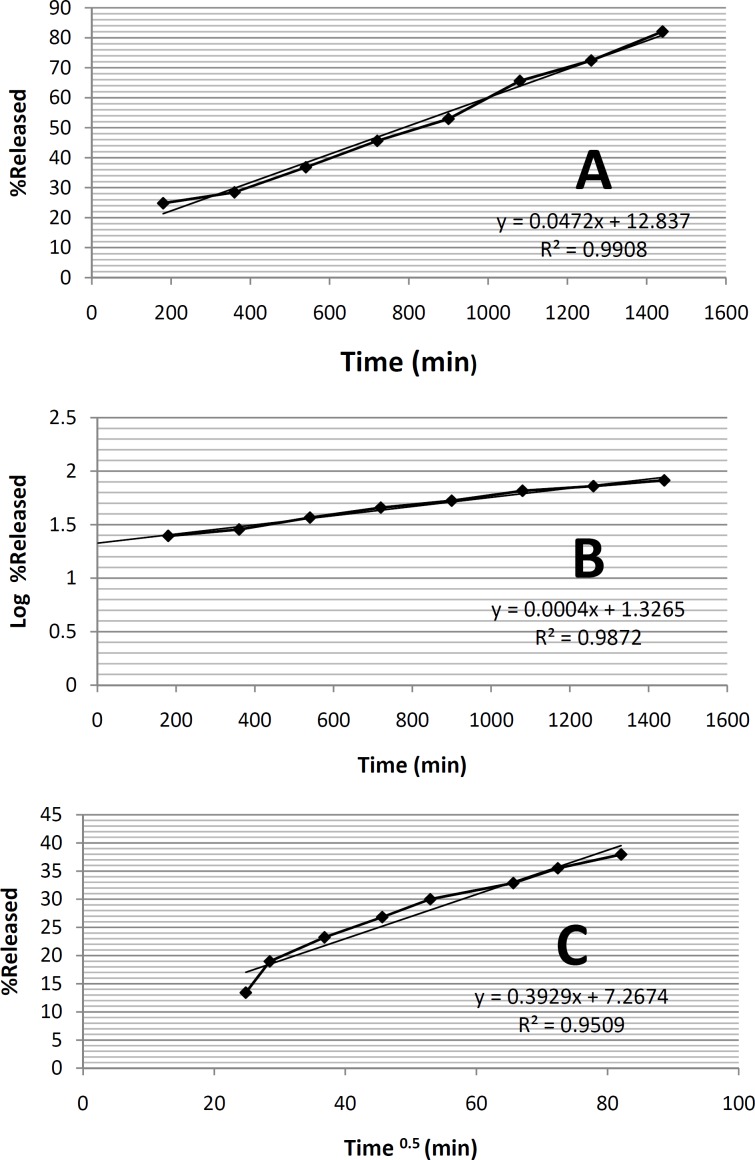
The *in-vitro *drug release kinetic studies on formulation ME-8a8. A. The mean of released percentage-time (Zero order kinetic); B. Logaritm of released percentage mean-time (first order kinetic); C. Released percentage mean-Square of time (Higuchi kinetic).

The percentage of released tretinoin was also calculated from commercial cream and gel containing the same amount of tretinoin that was respectively 45.56% and 65.7% after 24 h. The results obtained (Figure 2) showed that formulation ME-8a8 had the maximum amount of drug release, and 82.07% of its drug content was released after 24 h. This was the greatest among all formulations of gel and cream.

formulation ME-8a8, three mathematical models namely zero order kinetic, first order kinetic and higuchi model were investigated (Figure 3). In this selected microemulsion formulation prepared and underwent kinetic studies.

*In-vitro drug release kinetic studies on a selected formulation ME-8a8*

According to the results of the statistical analysis, in order to find out the mechanism of tretinoin release from the selected microemulsion formulation ME-8a8, three mathematical models namely zero order kinetic, first order kinetic and higuchi model were investigated (Figure 3). In this selected microemulsion formulation (ME-8a8), the calculated regression coefficients for zero order, first order and higuchi models were 0.9908, 0.9872, 0.9509 respectively. The model that best fitted the release data of tretinoin microemulsion formulation was evaluated by highest regression coefficient (R2).

Comparison of correlation coefficients showed that the kinetics of drug release from formulation ME-8a8 followed zero order model of kinetics (r2 = 0.9908).

It should be noted that the zero order kinetic model describes systems where the drug release rate is independent of its concentration. In contrast, first order kinetic model is indicative of systems where drug release is a concentration-dependent process. Higuchi’s model describes the release of drugs from formulation as the square root of a time dependent process, based on Fickian diffusion (34).

*Determining the rheological behavior of selected microemulsion*

**Figure 4:**
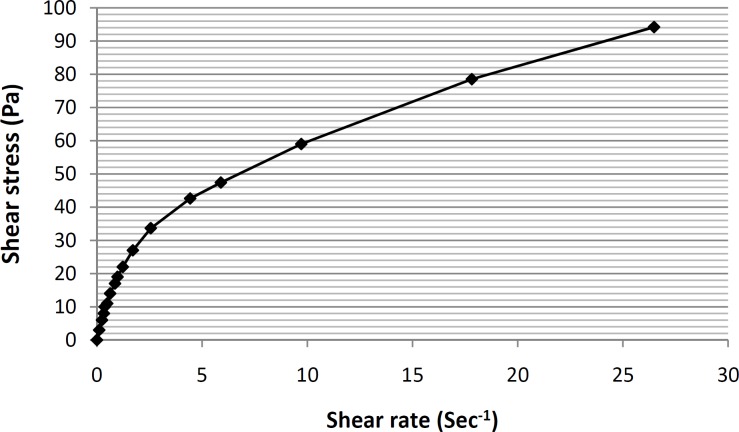
Rheogram of the formulation ME-8a8 formulation, showing the presence of a pseudoplastic behavior (n = 3,data points are presented as mean ± SD).

Viscosity and rheological behaviors are among the most important and noteworthy characteristics of any vehicle used for topical application within the hygienic/cosmetic field (35). The formulation of ME-8a8 viscosities was measured using Brookfield stainless steel cone/ plate viscometer. The rheogram of the selected formulation has been shown in Figure 4.

**Figure 5:**
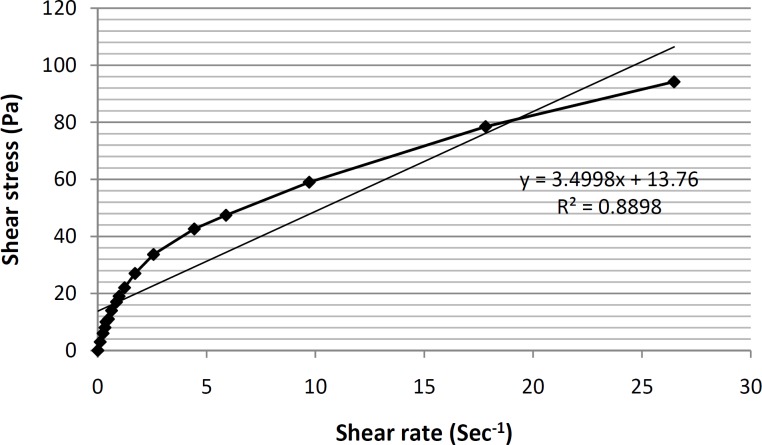
Rheogram of the formulation ME-8a8 formulation, showing the presence of a pseudoplastic behavior (n = 3, data points are presented as mean ± SD).

As it can be seen, based on the obtained rheogram, the curve begins at the origin consequently and no part of the curve is linear, which shows non-Newtonian behavior. Since, the viscosity decreases with increasing rate of shear (shear-thining system), selected formulation ME-8a8 showed the pseudoplastic rheological behavior. Linear regression analysis of the corresponding plots was R2 = 0.8898 (Figure 5).

**Figure 6:**
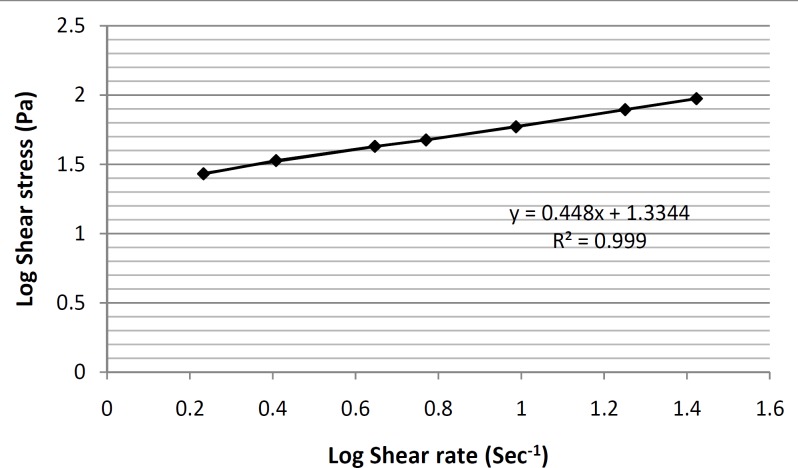
Log Shear stress-Log Shear rate. Rheogram of the formulation ME-8a8 formulation, showing the presence of a pseudoplastic behavior (n = 3, data points are presented as mean ± SD).

In order to make this non-linear region as linear as possible and determine the y-intercept more accurately, the log10 values of shear stress were plotted against the log10 values of shear rate (Figure 6).

**Figure 7:**
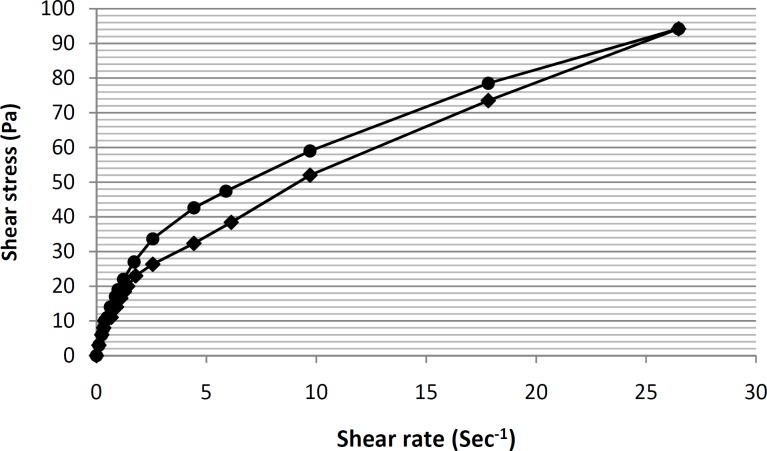
Rheogram of the formulation ME-8a8 formulation, showing the presence of a pseudoplastic thixotropic behavior (n = 3, data points are presented as mean ± SD).

In this way, a linear equation (y = 0.448x+ 1.3344) for this region was obtain, and by calculating the antilog of the y-intercepts (1.3344 in the equation), viscosity value was determined. Furthermore, the thixotropic behavior of selected formulation containing tretinoin-based microemulsion was also examined by constructing the appropriate up-curves and down-curves (Figure 7).

By constructing the up-curve and down-curve of formulation ME-8a8 (Figure 7), the resulting rheogram showed the presence of a pseudoplastic thixotropic behavior. This is a desirable behavior for topical drug delivery systems, since the formulation could remain stable following the preparation and packaging within the container, and then could break down as a result of shearing stresses applied during exit from the container and spreading on the skin surface (36).

## Conclusion

There were many parameters that could affect on drug release profiles from microemulsion bases, such as microemulsion type, viscosity, tretinoin solubility with different bases and ratio of surfactant/co-surfactant and oil phase. In this research, among two types of microemulsion bases, o/w microemulsions showed a better physicochemical properties and drug release profile, which can also have a suitable percutaneous absorption, by affecting on stratum corneum hydration. *In-vitro *evaluations, demonstrated that oil in water tretinoin microemulsion composed of 15% olive oil, 33% Tween^®^80, 12% propylene glycol and 40% distilled water could have a great potential for transdermal drug delivery.
